# 248. Evaluation of Continuous versus Intermittent Infusion of Nafcillin in the Treatment of Methicillin-Susceptible *Staphylococcus aureus* Bacteremia

**DOI:** 10.1093/ofid/ofad500.321

**Published:** 2023-11-27

**Authors:** Julie E Williamson, Sarah Wood, Travis J Carlson, Rupal K Jaffa, Michael S Boger, Michael S Boger

**Affiliations:** Atrium Health, Charlotte, North Carolina; Atrium Health Carolinas Medical Center, Charlotte, North Carolina; High Point University Fred Wilson School of Pharmacy, High Point, North Carolina; Atrium Health, Charlotte, North Carolina; Atrium Health, Charlotte, North Carolina; Atrium Health, Charlotte, North Carolina

## Abstract

**Background:**

Anti-Staphylococcal penicillins (ASPCN) are first-line agents in the treatment of methicillin-susceptible *Staphylococcus aureus* bacteremia (MSSA-B), and continuous infusion (CI) of oxacillin was associated with higher rates of 30-day microbiological cure versus intermittent (II) in infective endocarditis. Nafcillin (NAF) is the formulary ASPCN at Atrium Health (AH) and is variably ordered as CI or II. The purpose of this study is to compare NAF infusion strategies and related outcomes in the treatment of MSSA-B.

**Methods:**

This was a retrospective, observational, multisite study at 15 sites in the Greater Charlotte, NC Region of AH. Hospitalized adults with MSSA-B who received NAF from 1/1/2020 – 3/31/2022 were included. The primary outcome was receipt of CI vs II NAF. Secondary outcomes included time to microbiological cure (TTMIC), in-hospital 30-day all-cause mortality, hospital length of stay (LOS), and rates of adverse drug events (ADE) - acute kidney injury (serum creatinine increase at least 1.5 times baseline), transaminitis (aspartate aminotransferase / alanine transaminase ≥ 5 times the upper limit of normal), hypokalemia (potassium of ≤ 2.9 mmol/L), and allergic reaction. TTMIC, in-hospital mortality, LOS, and ADEs were also evaluated in a cohort of patients in the ICU within 48 hours of NAF start.

**Results:**

A total of 166 patients were included: 99 (59.6%) in the CI group and 67 (40.4%) in the II group. Thirteen patients in the II group were eventually changed to CI, but their results were analyzed in the II group. TTMIC was not significantly different between the CI and II group (5 vs 4 days, respectively p = 0.56), with similar time to receipt of NAF in both groups (Figure 1). In-hospital mortality, hospital LOS, and rates of ADE also did not differ between groups (Figure 1). Forty-seven patients were included in the ICU cohort: 29 in the CI group and 18 in the II group. Trends observed in the ICU cohort were similar to those observed in the larger population (Figure 2).

**Table 1.**
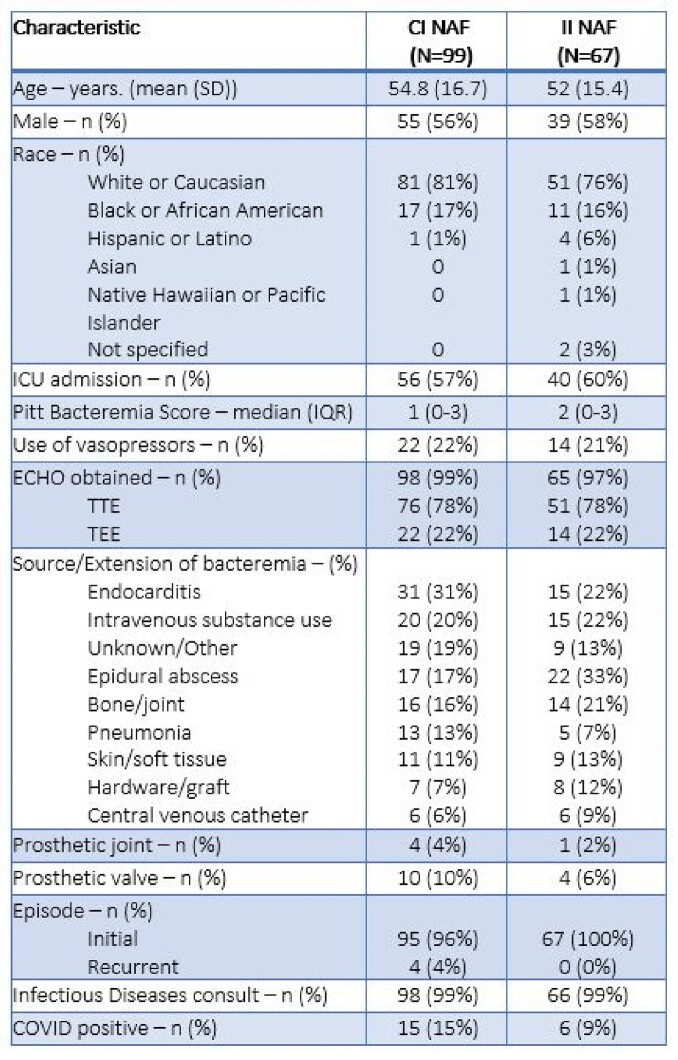
Selected Baseline Characteristics

Figure 1

**Figure:**
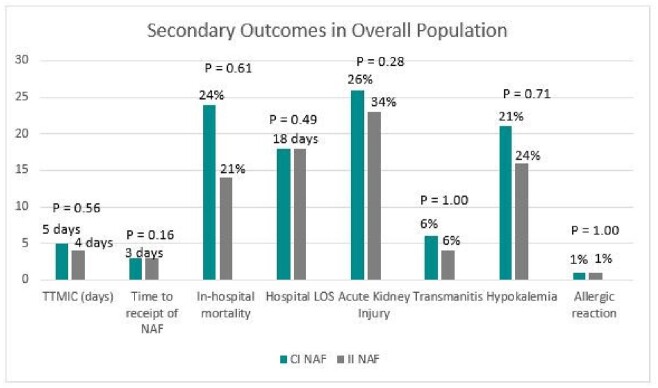

Figure 2

**Figure:**
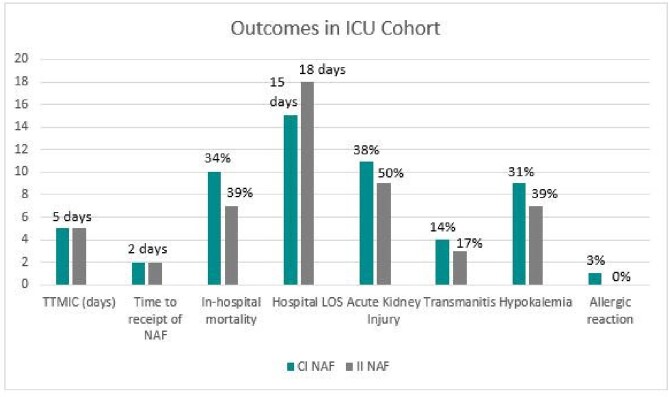

**Conclusion:**

In a small, unmatched, observational cohort of patients with MSSA-B, outcomes were not different based on NAF administration route. This suggests that administration strategy should be determined based on patient specific factors, such as available lines and patient convenience.

**Disclosures:**

**All Authors**: No reported disclosures

